# The interplay between mitochondrial DNA genotypes, female infertility, ovarian response, and mutagenesis in oocytes 

**DOI:** 10.1093/hropen/hoae074

**Published:** 2024-12-30

**Authors:** Annelore Van Der Kelen, Letizia Li Piani, Joke Mertens, Marius Regin, Edouard Couvreu de Deckersberg, Hilde Van de Velde, Karen Sermon, Herman Tournaye, Willem Verpoest, Frederik Jan Hes, Christophe Blockeel, Claudia Spits

**Affiliations:** Vrije Universiteit Brussel (VUB), Brussels Health Campus/Faculty of Medicine and Pharmacy, Research Group Genetics, Reproduction and Development , Laarbeeklaan 103, 1090 Brussels, Belgium; Universitair Ziekenhuis Brussel (UZ Brussel), Brussels Health Campus, Centre for Medical Genetics, Laarbeeklaan 101, 1090 Brussels, Belgium; Vrije Universiteit Brussel (VUB), Brussels Health Campus/Faculty of Medicine and Pharmacy, Research Group Genetics, Reproduction and Development , Laarbeeklaan 103, 1090 Brussels, Belgium; Universitair Ziekenhuis Brussel (UZ Brussel), Brussels Health Campus, Centre for Reproductive Medicine, Brussels IVF, Laarbeeklaan 101, 1090 Brussels, Belgium; Department of Clinical Sciences and Community Health, University of Milan, Milan, Italy; Infertility Unit, Fondazione IRCCS Ca’ Granda Ospedale Maggiore Policlinico, Milan, Italy; Vrije Universiteit Brussel (VUB), Brussels Health Campus/Faculty of Medicine and Pharmacy, Research Group Genetics, Reproduction and Development , Laarbeeklaan 103, 1090 Brussels, Belgium; Vrije Universiteit Brussel (VUB), Brussels Health Campus/Faculty of Medicine and Pharmacy, Research Group Genetics, Reproduction and Development , Laarbeeklaan 103, 1090 Brussels, Belgium; Vrije Universiteit Brussel (VUB), Brussels Health Campus/Faculty of Medicine and Pharmacy, Research Group Genetics, Reproduction and Development , Laarbeeklaan 103, 1090 Brussels, Belgium; Vrije Universiteit Brussel (VUB), Brussels Health Campus/Faculty of Medicine and Pharmacy, Research Group Genetics, Reproduction and Development , Laarbeeklaan 103, 1090 Brussels, Belgium; Universitair Ziekenhuis Brussel (UZ Brussel), Brussels Health Campus, Centre for Reproductive Medicine, Brussels IVF, Laarbeeklaan 101, 1090 Brussels, Belgium; Vrije Universiteit Brussel (VUB), Brussels Health Campus/Faculty of Medicine and Pharmacy, Research Group Genetics, Reproduction and Development , Laarbeeklaan 103, 1090 Brussels, Belgium; Vrije Universiteit Brussel (VUB), Brussels Health Campus/Faculty of Medicine and Pharmacy, Research Group Genetics, Reproduction and Development , Laarbeeklaan 103, 1090 Brussels, Belgium; Universitair Ziekenhuis Brussel (UZ Brussel), Brussels Health Campus, Centre for Reproductive Medicine, Brussels IVF, Laarbeeklaan 101, 1090 Brussels, Belgium; Vrije Universiteit Brussel (VUB), Brussels Health Campus/Faculty of Medicine and Pharmacy, Research Group Genetics, Reproduction and Development , Laarbeeklaan 103, 1090 Brussels, Belgium; Universitair Ziekenhuis Brussel (UZ Brussel), Brussels Health Campus, Centre for Reproductive Medicine, Brussels IVF, Laarbeeklaan 101, 1090 Brussels, Belgium; Department of Reproductive Medicine, Utrecht University Medical Centre, Utrecht, The Netherlands; Vrije Universiteit Brussel (VUB), Brussels Health Campus/Faculty of Medicine and Pharmacy, Research Group Genetics, Reproduction and Development , Laarbeeklaan 103, 1090 Brussels, Belgium; Universitair Ziekenhuis Brussel (UZ Brussel), Brussels Health Campus, Centre for Medical Genetics, Laarbeeklaan 101, 1090 Brussels, Belgium; Vrije Universiteit Brussel (VUB), Brussels Health Campus/Faculty of Medicine and Pharmacy, Research Group Genetics, Reproduction and Development , Laarbeeklaan 103, 1090 Brussels, Belgium; Universitair Ziekenhuis Brussel (UZ Brussel), Brussels Health Campus, Centre for Reproductive Medicine, Brussels IVF, Laarbeeklaan 101, 1090 Brussels, Belgium; Vrije Universiteit Brussel (VUB), Brussels Health Campus/Faculty of Medicine and Pharmacy, Research Group Genetics, Reproduction and Development , Laarbeeklaan 103, 1090 Brussels, Belgium

**Keywords:** mitochondrial DNA, heteroplasmy, homoplasmy, haplogroup, medically assisted reproduction (MAR), ovarian stimulation, infertility, oocyte

## Abstract

**STUDY QUESTION:**

Is there an association between different mitochondrial DNA (mtDNA) genotypes and female infertility or ovarian response, and is the appearance of variants in the oocytes favored by medically assisted reproduction (MAR) techniques?

**SUMMARY ANSWER:**

Ovarian response was negatively associated with global non-synonymous protein-coding homoplasmic variants but positively associated with haplogroup K; the number of oocytes retrieved in a cycle correlates with the number of heteroplasmic variants in the oocytes, principally with variants located in the hypervariable (HV) region and rRNA loci, as well as non-synonymous protein-coding variants.

**WHAT IS KNOWN ALREADY:**

Several genes have been shown to be positively associated with infertility, and there is growing concern that MAR may facilitate the transmission of these harmful variants to offspring, thereby passing on infertility. The potential role of mtDNA variants in these two perspectives remains poorly understood.

**STUDY DESIGN, SIZE, DURATION:**

This cohort study included 261 oocytes from 132 women (mean age: 32 ± 4 years) undergoing ovarian stimulation between 2019 and 2020 at an academic center. The oocyte mtDNA genotypes were examined for associations with the women’s fertility characteristics.

**PARTICIPANTS/MATERIALS, SETTING, METHODS:**

The mtDNA of the oocytes underwent deep sequencing, and the mtDNA genotypes were compared between infertile and fertile groups using Fisher’s exact test. The impact of the mtDNA genotype on anti-Müllerian hormone (AMH) levels and the number of (mature) oocytes retrieved was assessed using the Mann–Whitney *U* test for univariate analysis and logistic regression for multivariate analysis. Additionally, we examined the associations of oocyte maturation stage, infertility status, number of ovarian stimulation units, and number of oocytes retrieved with the type and load of heteroplasmic variants using univariate analysis and Poisson or linear regression analysis.

**MAIN RESULTS AND THE ROLE OF CHANCE:**

Neither homoplasmic mtDNA variants nor haplogroups in the oocytes were associated with infertility status or with AMH levels. Conversely, when the relationship between the number of oocytes retrieved and different mtDNA genotypes was examined, a positive association was observed between the number of metaphase (MII) oocytes (*P* = 0.005) and haplogroup K. Furthermore, the presence of global non-synonymous homoplasmic variants in the protein-coding region was significantly associated with a reduced number of total oocytes and MII oocytes retrieved (*P* < 0.001 for both). Regarding the type and load of heteroplasmic variants in the different regions, there were no significant associations according to maturation stage of the oocyte or to fertility status; however, the number of oocytes retrieved correlated positively with the total number of heteroplasmic variants, and specifically with non-synonymous protein-coding, HV and rRNA variants (*P* < 0.001 for all).

**LIMITATIONS, REASONS FOR CAUTION:**

The current work is constrained by its retrospective design and single-center approach, potentially limiting the generalizability of our findings. The small sample size for specific types of infertility restricts this aspect of the findings.

**WIDER IMPLICATIONS OF THE FINDINGS:**

This work suggests that mitochondrial genetics may have an impact on ovarian response and corroborates previous findings indicating that the size of the oocyte cohort after stimulation correlates with the presence of potentially deleterious variants in the oocyte. Future epidemiological and functional studies based on the results of the current study will provide valuable insights to address gaps in knowledge to assess any prospective risks for MAR-conceived offspring.

**STUDY FUNDING/COMPETING INTEREST(S):**

This work was supported by the Research Foundation Flanders (FWO, Grant numbers 1506617N and 1506717N to C.S.), by the Fonds Wetenschappelijk Fonds, Willy Gepts Research Foundation of Universitair Ziekenhuis Brussel (Grant numbers WFWG14-15, WFWG16-43, and WFWG19-19 to C.S.), and by the Methusalem Grant of the Vrije Universiteit Brussel (to K.S.). M.R. and E.C.d.D. were supported predoctoral fellowships by the FWO, Grant numbers 1133622N and 1S73521N, respectively. The authors declare no conflict of interests.

**TRIAL REGISTRATION NUMBER:**

N/A.

WHAT DOES THIS MEAN FOR PATIENTS? Some genetic variation is linked to infertility, raising concerns that fertility treatments could pass these harmful genetic changes to children, continuing the cycle of infertility. If changes in the mitochondrial DNA, a small genome specific to the cell’s energy producers, can play a similar role remains unclear. In this study, we investigated whether certain genetic variations in mitochondrial DNA are linked to female infertility. We also explored the link between mitochondrial DNA variation and outcomes of ovarian stimulation, a procedure that uses medication to help the ovaries mature multiple oocytes per menstrual cycle for assisted reproduction treatments. We first focused on different patterns of variations in the mitochondrial DNA that are shared by groups of individuals and inherited from a common ancestor; these groups of variants are called haplogroups. Additionally, we also studied other variants in the mitochondrial DNA that do not belong to any haplogroup.

The study analyzed genetic information in the oocytes of 132 women undergoing ovarian stimulation for IVF procedures between 2019 and 2020. Our results show that a specific mitochondrial DNA haplogroup, called haplogroup K, is associated with a better ovarian response, meaning women with this genetic variation tended to produce more mature oocytes upon hormonal stimulation. On the other hand, other genetic variations, specifically ones that affect the production of proteins, were linked to fewer numbers of oocytes retrieved. It was also interesting to note that the higher the number of oocytes retrieved in a cycle, the higher the number of specific genetic differences identified in the oocytes, suggesting a deleterious effect for strong ovarian stimulation.

Overall, this study sheds light on how mitochondrial genetics might influence fertility treatment outcomes and highlights the need for further research in this area to better understand the potential risks associated with medically assisted reproduction techniques.

## Introduction

For the last three decades, the field of biomedical research has been striving to understand the complex genetic factors associated with disease, and infertility has been no exception (Claussnitzer *et al.*, 2020). Infertility has been linked to an array of large and small chromosome abnormalities and to variants in genes involved in organogenesis, gametogenesis, and hormone homeostasis as well as other processes (Krausz and Riera-Escamilla, 2018; Yatsenko and Rajkovic, 2019). For example, a meta-analysis on genetic causes of female infertility identified 105 genes that play a role as monogenic causes of this phenotype (Van Der Kelen *et al.*, 2023), while a similar review identified 120 genes associated with male infertility (Houston *et al.*, 2021).

Bearing in mind the significant genetic component of infertility, there is the concern that medically assisted reproduction (MAR) may be favoring the transmission of these deleterious variants to the offspring, resulting in the inheritance of an infertility phenotype (Lipshultz and Lamb, 2007). For instance, we know that children born after MAR are at an increased risk of puberty disorders (Klemetti *et al.*, 2022), and men conceived by ICSI from fathers with low sperm counts have lower median sperm concentrations, lower total sperm counts, and lower total motile sperm counts (Belva *et al.*, 2016). Furthermore, it is well-established that children conceived by MAR have a higher risk of being born with a congenital abnormality and of being small for gestational age (Qin *et al.*, 2017; Luke *et al.*, 2021), with an overall 2- to 3-fold increased risk of adverse perinatal outcomes (Pandey *et al.*, 2012; Pinborg *et al.*, 2013b; Qin *et al.*, 2017). Long-term follow-up studies show increasing evidence that these children are at risk of a spectrum of health issues later in life, including cardiovascular disorders and metabolic anomalies (Guo *et al.*, 2005; Ceelen *et al.*, 2008a,b; Lu *et al.*, 2013; Meister *et al.*, 2018; Valenzuela-Alcaraz *et al.*, 2019; Ahmadi *et al.*, 2023; Belva *et al.*, 2023).

Considering that we now know there is a significant genetic factor in infertility, it is interesting to note that one of the predictors of adverse neonatal outcomes is maternal subfertility, both after MAR and in naturally conceived children (Romundstad *et al.*, 2008; Middelburg *et al.*, 2010; Cooper *et al.*, 2011; Pinborg *et al.*, 2013a,b; Sunkara *et al.*, 2016, 2021; Yatsenko and Rajkovic, 2019; Houston *et al.*, 2021; Luke *et al.*, 2021). This is suggestive of the maternal genetic infertility background being inherited by newborns, manifesting as increased risks of adverse neonatal outcomes and other suboptimal health parameters later in life.

While genetic research in infertility has predominantly focused on the nuclear genome and on monogenic traits, mitochondrial DNA (mtDNA), due to its crucial role in health and its exclusively maternal inheritance, is a prime candidate for involvement in infertility (Bentov *et al.*, 2011; Zhang *et al.*, 2017; Derevyanko *et al.*, 2022; Kim *et al.*, 2022). The mtDNA comprises 16 569 base pairs and codes for 37 genes. A single cell contains numerous mitochondria, each carrying 2–10 copies of mtDNA (polyplasmy). Homoplasmy refers to the presence of a variant in all of the mtDNA molecules of an individual, whereas heteroplasmy indicates the presence of a variant in only some of the mtDNA molecules. The percentage of molecules carrying the variant is referred to as the heteroplasmic load. A combination of specific homoplasmic variants in the mtDNA inherited from a shared ancestor are called haplogroups.

The role of the mtDNA copy number has been studied both in oogenesis and embryonic development. For instance, the maintenance of a minimal threshold of mtDNA copies is pivotal for proper oocyte function and maturation (May-Panloup *et al.*, 2005a,b; Santos *et al.*, 2006), and this in turn has led to the idea that supplementation of mtDNA-deficient oocytes may improve not only fertilization rates but also blastocyst formation rates (St John *et al.*, 2016). In embryonic cells, the quantity of mtDNA has been suggested to predict the embryo’s quality, implantation capacity, and potential to result in a live birth (Cecchino and Garcia-Velasco, 2019).

Conversely, there are still very few studies providing insight on how mtDNA variation may impact male and female fertility or ovarian response during MAR. The mitochondrial macro-haplogroup JT has been suggested to have a protective effect in the context of diminished ovarian response (Gómez-Durán *et al.*, 2010; May-Panloup *et al.*, 2014). In men, haplogroups H, U, and T have been associated with reduced sperm motility (Ruiz-Pesini *et al.*, 2000; Montiel-Sosa *et al.*, 2006).

With regards to the association between infertility and mtDNA point mutations, close to no data is available. Variants in the mtDNA polymerase *POLG* may result in a more error-prone polymerase and *de novo* mtDNA mutagenesis, and this nuclear gene has been identified as an interesting candidate in the study of both male and female infertility (Demain *et al.*, 2017). Meta-analysis of genome-wide association studies has suggested an association between non-syndromic variants in *POLG* and the age of menopause (Stolk *et al.*, 2012), while other studies have found autosomal dominant *POLG* mutations associating with premature ovarian failure (Pagnamenta *et al.*, 2006) and with male infertility (Rovio *et al.*, 2001; Jensen *et al.*, 2004).

Recent work by our group established a functional link between heteroplasmic mtDNA variants and birthweight in naturally conceived and MAR-conceived children, with the latter more frequently carrying heteroplasmic mtDNA variants associated with lower birth weight (Mertens *et al.*, 2024). Notably, the frequency of these variants correlated with maternal age in both populations while in MAR-children, it also correlated with the number of oocytes retrieved after ovarian stimulation in the mother (Mertens *et al.*, 2024).

A relationship between maternal age and *de novo* mtDNA variants in offspring was previously identified by the study of naturally conceived mother–child pairs (Rebolledo-Jaramillo *et al.*, 2014; Zaidi *et al.*, 2019). On the other hand, ovarian stimulation may disrupt the selection of competent oocytes, potentially allowing the ovulation of oocytes with deleterious mtDNA variants (Floros *et al.*, 2018; Zhang *et al.*, 2018; Wei *et al.*, 2019; Zaidi *et al.*, 2019).

This study aimed at investigating the association between mtDNA variation and female infertility from multiple perspectives. First, we sought to study potential links between the mitochondrial haplogroups and other homoplasmic variants with ovarian reserve and response to ovarian stimulation. Second, we tested whether oocytes of infertile women more frequently transmitted the profile of specific variants previously associated with lower birthweights, as this hypothesis could not be tested using the cohort studied in our previous work (Mertens *et al.*, 2024). Lastly, we examined the relationship between ovarian response and the presence of heteroplasmic mtDNA variants in oocytes, seeking to corroborate our recently published results (Mertens *et al.*, 2024).

## Materials and methods

### Ethics

The data in this study were adequately protected and encrypted in compliance with the General Data Protection Regulation (GDPR). The Institutional Review Board of the Universitair Ziekenhuis Brussel (UZ BrusselB) granted approval for this study (B.U.N.: 143201939997). All participants signed to give informed consent.

### Study population

This was a single-center study at the UZ Brussel in Belgium, including data from women who underwent ovarian stimulation between 2019 and 2020. All women were aged between 18 and 42 years old at the time of ovarian stimulation. The ovarian stimulation procedure was conducted using two different protocols. The first protocol involved GnRH analogs to desensitize the pituitary gland, combined with either human menopausal gonadotrophins or recombinant FSH (long agonist protocol). The second protocol used a GnRH antagonist along with human menopausal gonadotrophins or recombinant FSH (short antagonist protocol). The initial dose of gonadotrophins was tailored based on the patient’s age, ovarian reserve markers, and previous response to ovarian stimulation. hCG or a GnRH analog was administered to trigger ovulation, once at least three follicles, with a mean diameter greater than 17 mm, were observed during a transvaginal ultrasound scan. The oocyte retrieval procedure was scheduled 36 h after hCG or GnRH analog administration (Verpoest *et al.*, 2008). The study population included women of both infertile or fertile couples. All women undergoing preimplantation genetic testing for monogenic disorders and those from couples exhibiting a distinct male infertility phenotype were designated as fertile on the condition that comprehensive fertility assessments yielded normal results (n = 93). Women with an identifiable cause of infertility were categorized as infertile (n = 4: 1 woman with polyps, 1 with Asherman Syndrome, 1 with microhyperprolactinemia, and 1 recurrent miscarriage). Women from couples without an identifiable cause of infertility were classified as idiopathic infertile (n = 35). An oocyte in metaphase II (MII) is considered a mature oocyte. Clinical information was extracted from the women’s medical records for female age, anti-Müllerian hormone (AMH), BMI, parity, cause of infertility, number of ovarian stimulation units (i.e. FSH units), number of oocytes retrieved, number of MII oocytes, and infertility status.

A total of 132 women were enrolled, contributing an average of 1.89 oocytes (SD 1.27) for mtDNA sequencing, with a total of 261 oocytes being analyzed. Unequivocal grading of the stage of maturation of the oocytes was available for 257 of the 261 oocytes. There were 53 mature oocytes (MII) and 204 immature oocytes, comprising 68 at the metaphase I (MI) stage and 136 at the germinal vesicle (GV) stage. The population had a mean female age of 32 years old (SD 4.4) with a normal ovarian reserve characterized by a mean AMH of 2.5 µg/l (SD 1.5). Baseline female and cycle characteristics are summarized in Table 1.

**Table 1.** Patient and cycle characteristics of the study population.

Patient characteristics	Mean (SD) or n (%)	Range
**Women (n)**	132	NA
**Female age (years)**	32.52 (±4.43)	21–41
**AMH (µg/l)**	2.49 (±1.53)	0.19–7.30
**BMI (kg/m²)**	23.82 (±4.43)	18–42
**Nulliparous**	104 (83.3%)	0
**Multiparous**	6 (4.5%)	2–3
**Female infertility**	4 (3.03%)	NA
**Female fertility**	93 (70.45%)	NA
**Idiopathic infertility**	35 (26.52%)	NA

**Cycle characteristics**	**Mean (SD) or n (%)**	**Range**

**Oocytes donated (n)**	261	
**Oocytes donated/woman (n)**	1.89 (±1.27)	1–7
**Stimulation units (IU)**	1861.05 (±754.08)	59.94–5115.00
**Oocytes retrieved/woman (n)**	12.13 (±6.68)	1–37
**MII oocytes retrieved/woman (n)**	8.96 (±5.03)	0–23

AMH, anti-Müllerian hormone; MII, metaphase II; NA, not appropriate.

### mtDNA sequencing

Oocytes were individually collected and lysed as previously described (Spits *et al.*, 2006). The methods for the sample preparation, sequencing, and data analysis have been extensively validated in house (Zambelli *et al.*, 2017) and detailed protocols can be found in Mertens *et al.*, (2019). In short, to enrich the samples for mtDNA, a long-range PCR was performed using the primer set (5042f-1424r): forward—5′AGC AGT TCT ACC GTA CAA CC-3′ and reverse—5′-ATC CAC CTT CGA CCC TTA AG-3′. This resulted in a 12.9 kb amplicon, which was subsequently subjected to massive parallel sequencing using the Illumina platform. The resulting sequences were aligned to the reference mitochondrial genome (NC_0.12920.1). The generated bam files were uploaded to the online platform ‘mtDNA server’ to determine the haplogroup and to identify other homoplasmic and heteroplasmic variants (Weissensteiner *et al.*, 2016) (https://mitoverse.i-med.ac.at/), and MuTect2 (Cibulskis *et al.*, 2013) was used to detect small insertions and deletions, while possible amino-acid changes were identified using MitImpact2 (Castellana *et al.*, 2015). Variants in regions of the mtDNA known to be susceptible to PCR and sequencing errors were excluded from the analysis. Variants present in 2–98% of the reads were considered heteroplasmic, while variants present in >98% of the reads were considered homoplasmic. Variants found in frequencies <2% were excluded to avoid the risk of false positive calling. The mtDNA variants were subsequently subdivided into different categories, depending on the location of the variation: the highly polymorphic hypervariable (HV) region, the non-coding (NonCod) region, the origin of replication on the heavy strand (OHR), the termination-associated sequence (TAS), the rRNA genes, the tRNA genes, and the protein-coding genes with synonymous (Syn) and non-synonymous (NonSyn) variants. These categories could be subdivided again into local and global variants. Local variants refer to variants that are described in the PhyloTree database, whereas global variants are variants that have not yet been reported.

### Statistics

The subjects’ characteristics are presented as numerical values and percentages, as well as either mean ± SD or median (minimum–maximum), as appropriate. A Fisher’s exact test with Bonferroni correction was performed to investigate the relationship between haplogroups and the presence of at least one homoplasmic variant and infertility status. Univariate linear regression analysis was performed to examine the relationship between female age and AMH, the number of oocytes, and the number of MII oocytes. A Mann–Whitney *U* test was preferred to examine the relationship between ovarian parameters (AMH, number of oocytes, number of MII oocytes) and haplogroups or the presence of at least one (global or local) homoplasmic variant. We considered parameters that showed a trend (with *P* < 0.2) toward affecting ovarian parameters in the subsequent multivariate logistic regression model. However, parameters that were rare in the study population (five or fewer occurrences) were not included in the multivariate logistic regression analysis. A chi-square test with Bonferroni correction was performed to investigate the relationship between the type and number of heteroplasmic variants and maturation stage and infertility. In addition, the relationship between the heteroplasmic load and the oocyte maturation stage and infertility was investigated using a Mann–Whitney *U* test with Bonferroni correction. A multivariate generalized Poisson loglinear regression model, corrected for female age was used to test the association between the type and number of heteroplasmic variants and the number of FSH units for ovarian stimulation as well as the number of oocytes retrieved. A multivariate linear regression analysis, corrected for female age was employed to test the association between the load of heteroplasmic variants and the number of FSH units for ovarian stimulation as well as the number of oocytes retrieved. AMH was not considered in those analyses, to avoid collinearity with female age. All statistical analyses were performed using SPSS IBM Statistics 28.0 software (Chicago, IL, USA).

## Results

### Homoplasmic variants and haplogroups do not associate with female infertility

**Figure 1.**
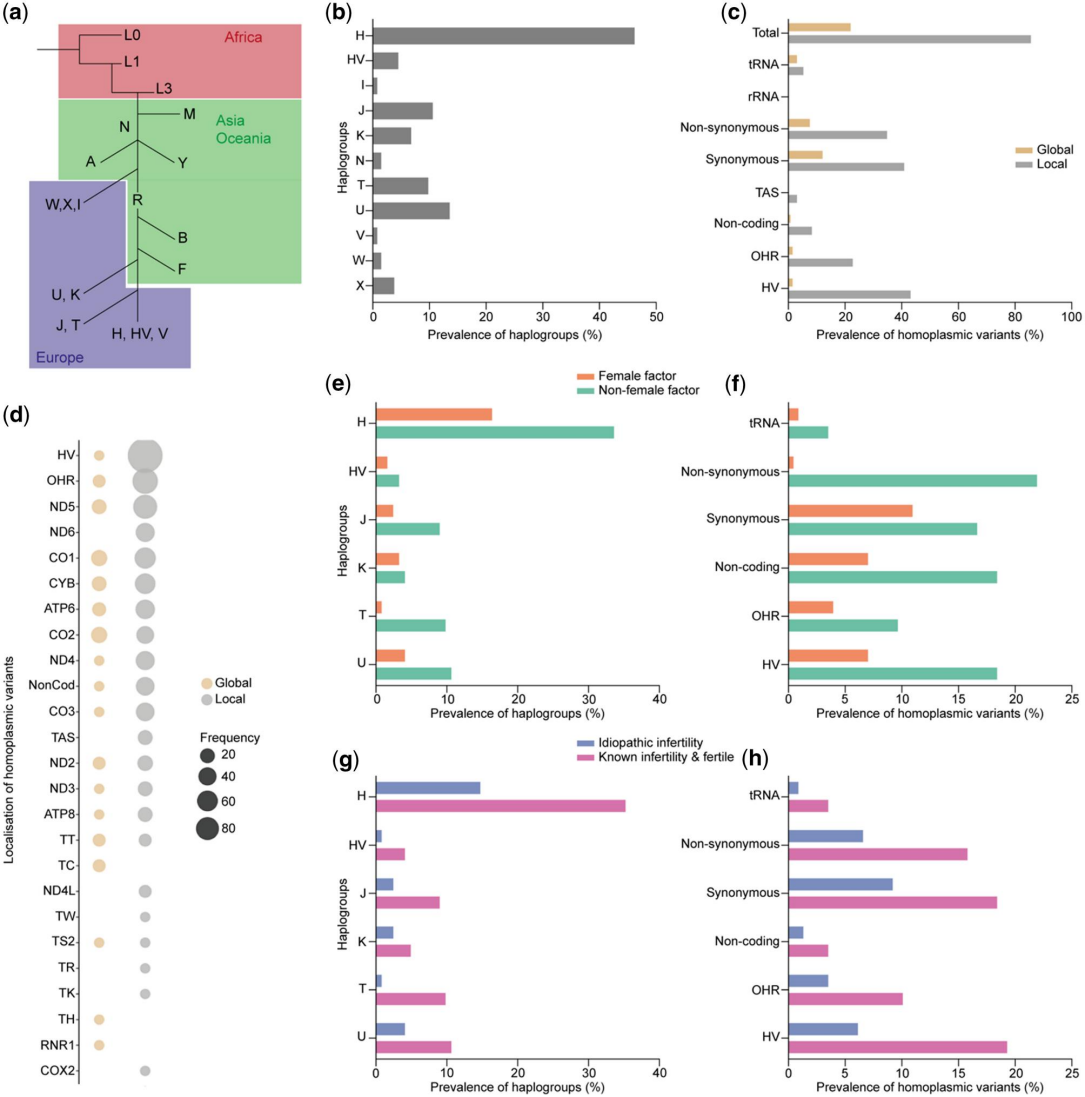
**Haplogroups and other homoplasmic mitochondrial DNA variants do not associate with female infertility**. (**a**) mitochondrial DNA (mtDNA) haplogroup tree, highlighting the origins of African, Asian/Oceanian, and European haplogroups. (**b**) Prevalence of the different haplogroups across the study population. (**c**) Prevalence of women carrying global and local homoplasmic variants, classified by location in the mtDNA and type. The percentages do not total 100% owing to the possibility of individuals having more than one variant. (**d**) Overview of all local and global homoplasmic variants, plotted according to their location in the mitochondrial genome. The size of the bubble is proportional to the variant’s frequency in the study population. (**e**) Prevalence of haplogroups in female factor infertility and non-female factor infertility. (**f**) Prevalence of women carrying homoplasmic variants located in the different regions of the mtDNA, categorized as female factor and non-female factor of infertility. (**g**) Prevalence of haplogroups in idiopathic infertility and known infertility or fertility. (**h**) Prevalence of women carrying homoplasmic variants located in the different regions of the mtDNA, categorized as idiopathic infertility and known infertility or fertility. Fisher’s exact test showed no significant differences across any of the analyses. HV, hypervariable region; NonCod, non-coding region; OHR, origin of replication on the heavy strand; TAS, termination-associated sequence.

First, we investigated the homoplasmic variants identified in the oocytes. This type of variant is always transmitted by a woman to her oocytes; hence, we assumed that any homoplasmic variant found in an oocyte will also be found in the corresponding woman. From these variants, we established the haplogroups, which relate to genetic ancestry and follow a regional distribution on our planet (Fig. 1a). The European haplogroups were most prevalent in our study population, with haplogroup H comprising most of the participants (46.2%), and haplogroups U and J representing 13.6% and 10.6% of women, respectively. Several other haplogroups were identified in the study population, albeit at lower percentages (Fig. 1b). Next, we categorized the homoplasmic variants that did not belong to the haplogroup of the women, according to their location in the mitochondrial genome: as ‘local’ when they have been reported to be associated with another haplogroup, or as ‘global’ when they were unique to this individual (Fig. 1c and d).

A substantial number of women carried homoplasmic variants in the HV region (n = 58, 43.9%), with the majority being local (n = 57, 43.2%) rather than global variants (n = 2, 1.5%). Among the participants, 11 women (8.3%) carried NonCod variants, with a predominant occurrence of local variants. OHR variants were identified in 31 women (23.5%), primarily local variants (n = 30, 22.7%). Only one local variant in the TAS region was detected (0.8%). tRNA variants were observed in 10 women (7.6%), consisting of 4 global (3.0%) and 7 local (5.3%) variants. The distribution of Syn and NonSyn variants showed a similar distribution between global and local variations (Syn: global = 16 (12.1%), local = 54 (40.9%); NonSyn: global = 11 (8.3%), local = 46 (34.9%)). Variants were observed across all 13 protein-coding genes, most commonly occurring in the *ATP6* (5.0%), *CO1* (7.6%)*, CO2* (5.0%)*, CO3* (3.8%), *CYB* (6.3%)*, ND4* (4.1%)*, ND5* (10.1%), *and ND6* (3.8%) genes. No rRNA variants were detected (Fig. 1c and d).

First, we checked whether the homoplasmic variants and haplogroups were linked to the infertility status of the women. For this, we categorized each patient as female factor infertility (including female (n = 4) and idiopathic infertility (n = 35)) vs non-female factor infertility (including male infertility (n = 39) and fertile couples (n = 54)). We hypothesized that, in the absence of male infertility, idiopathic cases could be considered as female infertility. A secondary categorization was also performed, comparing idiopathic infertility to both known infertility (including both female and male infertility) and fertile couples. This additional categorization aimed to explore whether mtDNA variations may associate with female infertility cases when a specific reason for infertility is excluded (i.e. idiopathic infertility). Figure 1e–h illustrate the distribution of various haplogroups and homoplasmic variations within the fertile and infertile study groups. No significant association was identified between local or global homoplasmic variations, nor haplogroup, and fertility status (using Fisher’s exact test with Bonferroni correction).

### The influence of mtDNA variation on AMH

**Figure 2.**
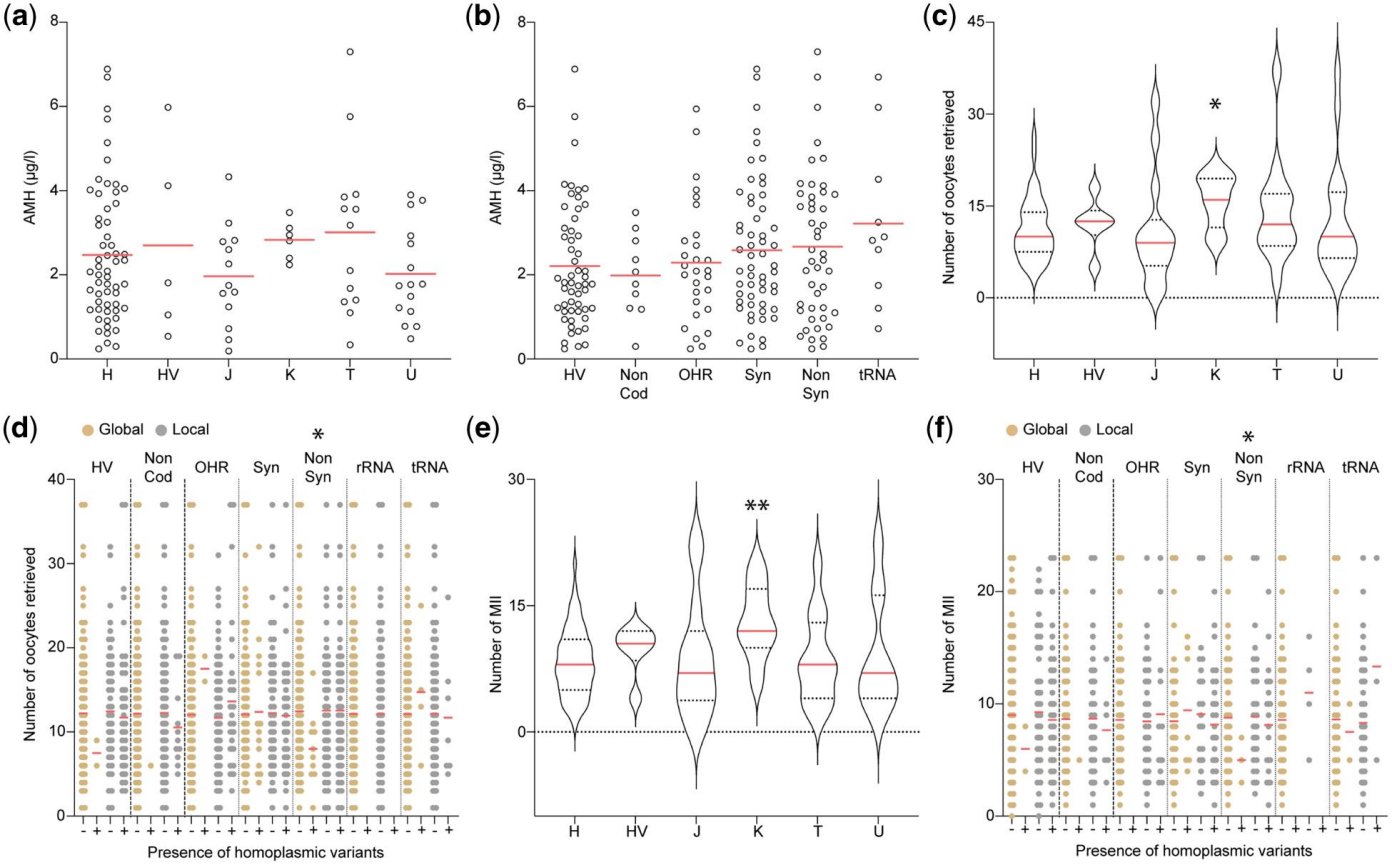
**Association of haplogroups and homoplasmic mitochondrial DNA variants to ovarian reserve and response to hormonal stimulation**. (**a**) The AMH levels of the women presented per haplogroup (non-significant differences, Mann–Whitney *U* test). (**b**) The AMH levels of the women categorized depending on the presence of homoplasmic variants in the different categories (non-significant differences, Mann–Whitney *U* test). (**c**) Number of oocytes retrieved per haplogroup (*P = 0.016, Mann–Whitney *U* test). (**d**) Number of oocytes retrieved in women carrying homoplasmic variants in the different categories (*P = 0.045, Mann–Whitney *U* test). (**e**) Number of MII oocytes retrieved per haplogroup (**P = 0.008), Mann–Whitney *U* test. (**f**) Number of MII oocytes retrieved in women carrying homoplasmic variants in the different categories (*P = 0.017, Mann–Whitney *U* test). In all plots, the mean is indicated with a red line. AMH, anti-Müllerian hormone; MII, metaphase II; HV, hypervariable region; NonCod, non-coding region; OHR, origin of replication on the heavy strand; TAS, termination-associated sequence; Syn, synonymous variant in protein-coding genes; NonSyn, non-synonymous variant in protein-coding genes.

Next, we studied whether there was an association between the mitochondrial genotype and AMH blood levels as a proxy for ovarian aging. Univariate linear regression analysis for the AMH levels of the women identified an expected significant negative association with female age (*P* < 0.001), but no other statistically significant associations were found when testing for the diverse haplogroups or the presence of homoplasmic variants across the different mitochondrial regions (Mann–Whitney *U* test, Fig. 2a and b). Subsequent assessment through multivariate linear regression, including all variables that had a *P* < 0.2 in the univariate analysis, confirmed the negative correlation between female age and AMH levels (*P* < 0.001), but did not reveal any further associations (Table 2).

**Table 2.** Multivariate regressions between mitochondrial DNA variation and anti-Müllerian hormone levels and ovarian response.

	B	95% CI for B	Significance
**AMH**			
Female age	−0.107	−0.166; −0.049	**<0.001**
Local HV homoplasmy	−0.456	−0.972; 0.059	0.083
**Total number of oocytes**			
Female age	−0.040	−0.051; −0.029	**<0.001**
Haplogroup K	0.110	−0.070; 0.290	0.233
Haplogroup J	−0.116	−0.335; 0.014	0.071
Local HV homoplasmy	−0.097	−0.202; 0.008	0.070
Global non-synonymous homoplasmy	−0.472	−0.686; −0.259	**<0.001**
**MII oocytes**			
Female age	−0.039	−0.052; −0.026	**<0.001**
Haplogroup K	0.279	0.085; 0.473	**0.005**
Global non-synonymous homoplasmy	−0.542	−0.800; −0.284	**<0.001**

*P*-values <0.05 are presented in bold. All multivariate regression models had an omnibus significance <0.001 and only included variables with *P* < 0.2 after univariate analysis. AMH, anti-Müllerian hormone; HV, hypervariable region; MII, metaphase II.

### Number of oocytes retrieved correlates negatively with the presence of patient-specific non-synonymous protein-coding homoplasmy and positively with haplogroup K

In the following analyses, we carried out univariate analysis to examine the relationship between the number of oocytes retrieved and different mtDNA genotypes. Linear regression showed a statistically significant negative correlation between female age and both the total number of retrieved oocytes and the number of MII oocytes (*P* < 0.001 for both). A positive association was observed between the total number of oocytes and MII oocytes and haplogroup K (Mann–Whitney *U* test, *P* = 0.016 and *P* = 0.008, respectively; Fig. 2c and e) and a significant negative correlation was identified between the presence of global non-synonymous variants in the protein-coding region and both the total number of oocytes and MII oocytes (Mann–Whitney *U* test, *P* = 0.045 and *P* = 0.017, respectively; Fig. 2d and f).

To further explore these associations and their potential interactions, we carried out linear regression for the total number of retrieved oocytes and the number of MII oocytes, including all variables that exhibited a *P* < 0.2 in the univariate analysis in the model. This confirmed the negative associations between the total number of retrieved oocytes and MII oocytes with female age (*P* < 0.001 for both; Table 2), the positive correlation between haplogroup K and the number of MII oocytes (*P* = 0.005), and the negative effect of the presence of global non-synonymous protein-coding variants for both the total number of retrieved oocytes and the number of MII oocytes (*P* < 0.001 for both; Table 2).

### Neither the maturation stage of the oocyte nor a background of infertility correlates with the type and load of heteroplasmic mtDNA variants

**Figure 3.**
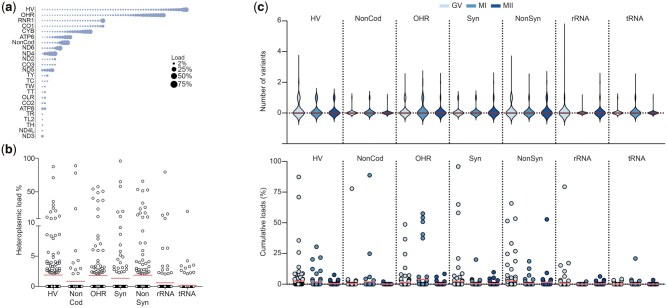
**The maturation stage of oocytes does not influence the number and load of heteroplasmic variants in the oocyte**. (**a**) Overview of all heteroplasmic variants identified, plotted according to their location in the mitochondrial genome. The size of the bubble is proportional to the variant’s frequency in the study population. The detection threshold was 2%. (**b**) Overview of the load of all heteroplasmic variants identified, categorized per region. The red line indicates the mean load. (**c**) Distribution of the number and load of heteroplasmic variants across the different stages of oocyte maturation. HV, hypervariable region, NonCod, non-coding region; OHR, origin of replication on the heavy strand; Syn, synonymous variant in protein-coding genes; NonSyn, non-synonymous variant in protein-coding genes; GV, germinal vesicle; MI, metaphase I; MII, metaphase II.

Next, we analyzed the heteroplasmic variants found in each of the sequenced oocytes. Heteroplasmic variants were predominantly observed in the HV region (n = 50), while none were observed in the TAS region. These heteroplasmic variations were also found across other regions such as NonCod (n = 11), OHR (n = 37), Syn (n = 24), NonSyn (n = 38), rRNA (n = 15), and tRNA (n = 12). The highest mean load of heteroplasmic variants was observed for variants in the HV and OHR region, as well as for NonSyn variations in the protein-coding region (all 1.8%). Syn variants appeared at a mean load of 1.3%, NonCod variants at 0.8% and variants in rRNA and tRNA regions at 0.6% and 0.2% respectively (Fig. 3a and b).

The study included 198 sibling oocytes, donated by the same donor. These oocytes carried 162 heteroplasmic variants, of which 153 heteroplasmic variants were unique to one oocyte, and only 9 heteroplasmic variants were observed in at least two sibling oocytes. The lack of somatic tissues to compare to made it impossible to reliably categorize the variants as inherited or *de novo*, and we proceeded without further considering this aspect.

**Figure 4.**
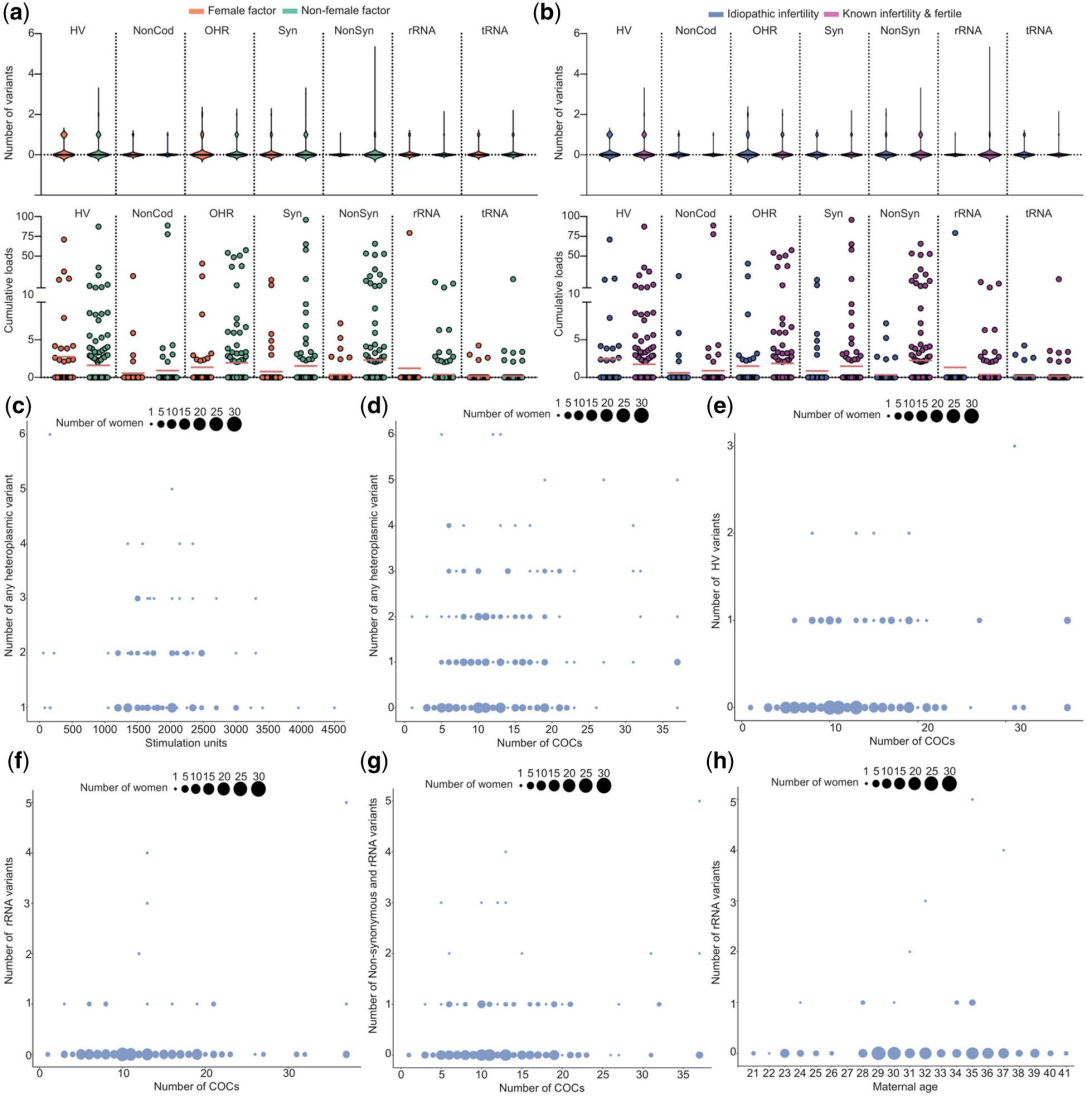
**The number of oocytes retrieved after ovarian stimulation influences the number of heteroplasmic variants in the oocyte**. (**a**) Distribution of the number and load of heteroplasmic variants in female factor and non-female factor infertility. The chi-square and the Mann–Whitney *U* analyses show no significant difference. (**b**) Distribution of the number and load of heteroplasmic variants in idiopathic infertility and known infertility or fertility. The chi-square and the Mann–Whitney *U* analyses show no significant difference. (**c**) Number of heteroplasmic variants per oocyte as a function of the total amount of follicle-stimulating hormone (FSH) units. The Poisson loglinear regression shows no significant effect. (**d**) Total number of heteroplasmic variants per oocyte as a function of the number of oocytes retrieved. The Poisson loglinear regression shows no significant effect. (**e**) Number of hypervariable region heteroplasmic variants per oocyte as a function of the number of oocytes retrieved. The Poisson loglinear regression shows a positive correlation (*P* < 0.001). (**f**) Number of rRNA heteroplasmic variants per oocyte as a function of the number of oocytes retrieved. The Poisson loglinear regression shows a positive correlation (<0.001). (**g**) The number of non-synonymous and rRNA heteroplasmic variants per oocyte as a function of the number of oocytes retrieved. The Poisson loglinear regression shows a positive correlation (*P*=0.002). (**h**) Number of rRNA heteroplasmic variants per oocyte as a function of the maternal age. The Poisson loglinear regression shows a positive correlation (*P*=0.03). Cumulative load refers to the sum of the heteroplasmic loads of multiple variants co-existing in one sample. HV, hypervariable region, NonCod, non-coding region; OHR, origin of replication on the heavy strand; Syn, synonymous variant in protein-coding genes; NonSyn, non-synonymous variant in protein-coding genes; COC, cumulus–oocyte complex.

First, we tested whether the oocyte maturation stage was associated with specific variants, and found no statistically significant differences in the type, number nor heteroplasmic load of the variants across GV, MI, and MII oocytes (Fig. 3c). Next, we studied whether the type, number, or load of heteroplasmic variants in the retrieved oocytes was associated with the women’s infertility status. Also here, we found no significant differences in the type, number, and the load of heteroplasmic variants in the different regions for female factor infertility and idiopathic infertility (Fig. 4a and b).

### Maternal age and the number of oocytes retrieved correlates with the number and type of heteroplasmic variants found in the oocytes

We next tested the effect of the total number of FSH units used during the ovarian stimulation, the maternal age, and the number of oocytes retrieved on the type and number of heteroplasmic variants found in the oocytes. AMH levels and duration of the ovarian stimulation were not included in the models because the first is strongly associated with maternal age, and the second with total FSH units. We carried out Poisson loglinear regressions for the number of variants identified in the different regions of the mtDNA per oocyte, and linear regressions for the heteroplasmic loads of the same variants.

**Figure 5.**
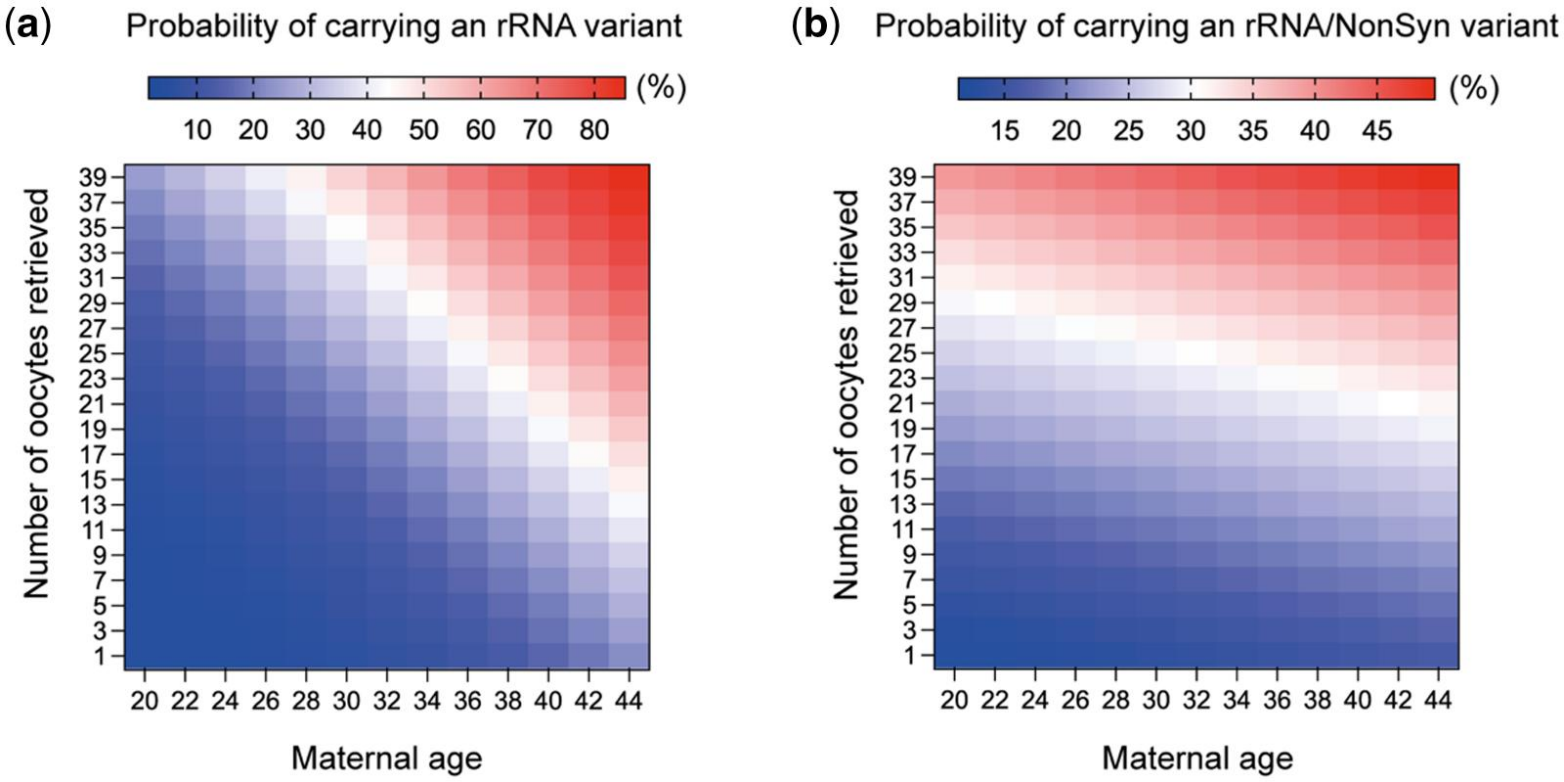
**Heatmap illustrating the probabilities of carrying a heteroplasmic variant in an oocyte retrieved after ovarian stimulation**. (**a**) Heatmap depicting the probability of carrying a heteroplasmic rRNA variant in an oocyte retrieved after ovarian stimulation, based on maternal age and the number of oocytes retrieved after ovarian stimulation. (**b**) A heatmap depicting the probability of carrying a heteroplasmic variant in either the rRNA region or a NonSyn variant in the protein-coding region, in an oocyte retrieved after ovarian stimulation and based on maternal age and the number of oocytes retrieved. Color-coding ranges from blue (lower probability) to red (higher probability). NonSyn, non-synonymous variant in protein-coding genes.

We did not identify any significant association between the total number of FSH units used during the ovarian stimulation and either the occurrence or the load of heteroplasmic variants in oocytes collected (Fig. 4c and Supplementary Tables S1 and S2). Conversely, there was a significant positive correlation between the number of retrieved oocytes and the total number of heteroplasmic variants, as well as for the number of heteroplasmic variants in the HV and rRNA regions and (*P* < 0.001 for both, Fig. 4d–f, Table 3, Supplementary Table S1). Additionally, a positive correlation was observed between the number of retrieved oocytes and the combination of rRNA and NonSyn variants in the protein-coding region (*P* = 0.01) (Fig. 4g, Table 3, Supplementary Table S1). We also found that female age positively correlated with the presence of rRNA variants in the oocytes (*P* = 0.03, Fig. 4h, Table 3, Supplementary Table S1). However, no correlation was observed between the heteroplasmic load itself and the number of oocytes retrieved or female age (Supplementary Table S2). Based on these regression models, we generated heatmaps to visualize the relationship between maternal age and the number of oocytes retrieved after ovarian stimulation, with the probability that an oocyte from that cycle carries an heteroplasmic rRNA variant or an RNA and/or a NonSyn variant (Fig. 5a and b, Supplementary Fig. S1).

**Table 3.** Generalized Poisson loglinear regression to predict the number of heteroplasmic variants in the oocytes.

	B	95% CI for B	Significance	Omnibus significance
**Total number **				**0.04 **
Female age	0.021	−0.011; 0.053	0.20	
Stim. Units	−0.00008	0.000; 0.000	0.48	
COC	0.0081	0.017; 0.049	**<0.001**	
**HV **				**0.04 **
Female age	−0.012	−0.074; 0.049	0.69	
Stim. Units	0.000	0.000; 0.001	0.33	
COC	0.050	0.021; 0.080	**<0.001**	
**rRNA **				**<0.001 **
Female age	0.121	0.013; 0.229	**0.03 **	
Stim. Units	0.000	−0.001; 0.000	0.11	
COC	0.080	0.041; 0.118	**<0.001 **	
**NonSyn+rRNA **				**0.02 **
Female age	0.017	−0.050; 0.344	0.55	
Stim. Units	0.000	0.000; 0.000	0.93	
COC	0.043	0.016; 0.070	**<0.01**	

The generalized Poisson loglinear regression model includes the female age, the total FSH stimulation units, and the number of oocytes retrieved to predict the total number of heteroplasmic variants, and HV, rRNA, and NonSyn+rRNA variants. *P*-values <0.05 are presented in bold. Stim.Units, stimulation units; HV, hypervariable region; Syn, synonymous variant in protein-coding genes; NonSyn, non-synonymous variant in protein-coding genes; COC, cumulus–oocyte complexes retrieved.

## Discussion

In the first part of this study, we investigated the relationship between mtDNA variation and female fertility, including the association of mitochondrial haplogroups and homoplasmic variants, with infertility status, ovarian reserve, and response to ovarian stimulation.

Haplogroups have been broadly studied in association with health status, including neurodegenerative, endocrine, and cardiovascular disorders (Hernández, 2023). In the field of reproductive medicine, haplogroup JT was identified as a potential marker for favorable ovarian aging owing to its decreased prevalence in women with low ovarian reserve parameters, such as AMH and antral follicle count (May-Panloup *et al.*, 2014). In this study, we found no association between haplogroups or other homoplasmic variants and female infertility, nor with the patients’ AMH levels. This was also the case when pooling the data for haplogroups J and T and categorizing the patients equivalently to the May-Panloup *et al.* study. This lack of association may be caused by underpowering due to an insufficiently accurate characterization of the infertility phenotype in our study population. Furthermore, the discrepancies with the results of May-Panloup *et al.* (2014) may also be caused by other genetic regulators impacting ovarian aging: e.g. the epigenetic clock (Hanson *et al.*, 2020; Li Piani *et al.*, 2022).

Our work did reveal an association between mtDNA genotypes and the outcomes of ovarian stimulation. Haplogroup K was positively associated with higher numbers of (MII) oocytes retrieved, while the presence of global non-synonymous variants in the protein-coding region was negatively associated with oocyte yield. This is in line with a previous study by Kumar *et al.* (2021) that also found that the total number of oocytes retrieved was highest for women with the haplogroups U–K; however, this association was not sustained when looking at MII oocytes. In our study, we also found that the presence of non-synonymous homoplasmic variants in the protein-coding region may be deleterious for the process of oocyte maturation, associating with a lower oocyte yield. The identified variants were specific to each patient, and do not appear in other haplogroups, which could be of interest to studies of etiology of reduced ovarian response in certain families. Taken together, while these findings suggest that mitochondrial genetic factors may influence fertility treatment outcomes, further research is needed to elucidate the underlying mechanisms driving these associations and their clinical implications for MAR.

In the second part of the study, we corroborated the recently identified influence of ovarian stimulation and maternal age on number of *de novo* mtDNA heteroplasmic variants in oocytes (Mertens *et al.*, 2024). There are two significant differences with our previous study. First, the previous study included only oocyte donors, while this study also includes women seeking MAR for different indications. Second, in this setup, we could not distinguish between inherited and *de novo* variants, because we did not have other patient tissues to compare with. In our previous study, 11% of the detected variants were inherited. Extrapolating to the current work, it is likely that the same fraction of the identified variants is inherited, although it does not appear to be sufficient to mask the effect of age and ovarian stimulation, independent of the gonadotrophin dose administered.

With regards to the origin of these variants, while the years-long residence of the oocytes in an ovary may be responsible for the age-dependent effect, we hypothesize that recruiting multiple follicles simultaneously disrupts the physiological selection of the ‘most fit’ oocytes (follicular dominance) and the degradation of lower-quality oocytes (follicular atresia), thereby also compromising the selection against oocytes with damaging mtDNA variants (Floros *et al.*, 2018). We observed an association between the number of oocytes retrieved after ovarian stimulation and the presence of heteroplasmic variants, but not with the heteroplasmic load of the variants. This observation could be due to certain variants exerting their effects regardless of their load, suggesting that even low levels of specific variants might significantly influence the outcome, while higher levels might not have a proportional impact. This has also been observed in a previous study, where even a low heteroplasmic load, below the conventional threshold for clinical impact, tends to be eliminated in human oocytes (Floros *et al.*, 2018).

Given the known pathogenic risk of certain heteroplasmic variations and their association with up to 20% of human disease, these findings pose a significant challenge (Ye *et al.*, 2014; Wilson *et al.*, 2016). We currently know little about the potential impact of this type of non-disease-associated variants on the individuals born from these oocytes, with work in mice suggesting a link to premature aging and infertility (Keogh and Chinnery, 2013; Ross *et al.*, 2014), and our recent work finding an association with lower birthweight (Mertens *et al.*, 2024). While this work confirms a link between ovarian stimulation and aging and the presence of particular variants, further research is required to prove causality in any potential clinical implications.

Our data give the opportunity for at least two reflections. First, mitochondria matter in reproductive science: some mitochondrial genotypes appear to have a role in response to ovarian stimulation. Our results add to the foundation of previous findings that postulate that mitochondrial quality influences the processes of oogenesis and oocyte maturity, corroborating their interest as a potential therapeutic target (Ben-Meir *et al.*, 2015; Sreerangaraja Urs *et al.*, 2020; Kirillova *et al.*, 2021; Zhou *et al.*, 2022). However, further research is first required to understand the mechanistic link. Second, less is more. The concern that an excessive number of oocytes retrieved could be detrimental for oocyte and embryonic quality has been voiced in the past (Santos *et al.*, 2010; Bosch *et al.*, 2016). While work in mice and cows has shown dose-dependent deleterious effects of ovarian stimulation (Ertzeid and Storeng, 2001; Van der Auwera and D’Hooghe, 2001; Chao *et al.*, 2005; Edwards *et al.*, 2005; Lee *et al.*, 2005; Ge *et al.*, 2012), it is still unclear if this is also true for humans. In this study and our previous work, we have observed that the protective mitochondrial bottleneck selection may be lost during ovarian stimulation with higher oocyte yields, increasing the number of potentially deleterious variants in the oocytes.

Translating our results into clinical practice is a complex issue. The ‘right’ number of oocytes is likely to be not the same for all but tailored to be patient specific. This perspective is especially true for patients with advanced maternal age, where, in order to compensate for the poorer prognosis, efforts are often made to increase the number of oocytes obtained. If we consider the usual clinical prediction model adopted to estimate the number of mature oocytes required for at least one euploid embryo, i.e. the MAR calculator, in the case of women aged 39 and 40 years, 16 and 19 oocytes are required, respectively (Esteves *et al.*, 2019). However, in this range of age and oocytes retrieved, the likelihood for an oocyte to carry a potentially harmful heteroplasmic variant is an estimated 1.5 times higher than that found in the general population (25% vs 15.6%) (Wei *et al.*, 2019). Considering that these variants are associated with lower birthweight (Mertens *et al.*, 2024), and may be linked to poorer health outcomes in the future (von Kleist-Retzow *et al.*, 2003; Montiel-Sosa *et al.*, 2006; Gibson *et al.*, 2008; Schon *et al.*, 2012; Ebner *et al.*, 2015; Stewart and Chinnery, 2015), the potential risk of underestimation of the detrimental role of ovarian stimulation in this population with poorer prognosis should be carefully considered.

Finally, there are several limitations to this study, which mainly stem from the sample size used. First, the phenotype–genotype associations may be sensitive to the sample size we achieved. This is particularly relevant to the haplogroup associations, which are known to be often difficult to replicate. Second, for the regression models to be usable in a clinical setting, we would require a much higher data density, particularly at the youngest and oldest age ranges and at the lowest and highest numbers of oocytes retrieved. The model now assumes a linear association between age, oocytes collected, and the chance of carrying a heteroplasmic variants, which is likely only correct for the mid-range of the data. This is obvious from the fact that while the model adjusts well to the known incidences of rRNA and non-synonymous protein-coding variants in the general population (1.7% for rRNA, 15.6% for rRNA and non-synonymous) (Wei *et al.*, 2019), the upper values in the rRNA regression model are excessively high, and higher than the equivalent coordinates in rRNA and non-synonymous model. In this light, our work should be considered as a starting point to design a large-scale study to build predictive models in a clinical setting. Third, the poor clinical characterization of the female infertility phenotype in our dataset limited the possibility to identify any associations with mtDNA genetic variation. Finally, the limited number of cases using an agonist protocol restricted our ability to perform robust statistical comparisons between the two main stimulation protocols (i.e. agonist and antagonist protocols). Consequently, we were unable to draw definitive conclusions about the impact of protocol type on oocyte retrieval outcomes based on the available data.

In sum, this study provides further insights into the complex interplay between mtDNA variation and infertility. We found indications that specific homoplasmic variants may impact ovarian response and confirmed our previously identified association between ovarian stimulation and age and the presence of heteroplasmic mtDNA variants in the oocytes retrieved. Overall, we hope our work will encourage broader research on the mitochondrial genetic component in reproductive medicine.

## Supplementary Material

hoae074_Supplementary_Data

## Data Availability

The source data for each figure panel in the article, as well as the data underlying the analysis of the study can be found in the Open Science Framework, at https://osf.io/upd2c/. The raw sequencing data are available upon request.
