# Correction to: The interplay between mitochondrial DNA genotypes, female infertility, ovarian response, and mutagenesis in oocytes

**DOI:** 10.1093/hropen/hoaf037

**Published:** 2025-07-07

This is a correction to: Annelore Van Der Kelen, Letizia Li Piani, Joke Mertens, Marius Regin, Edouard Couvreu de Deckersberg, Hilde Van de Velde, Karen Sermon, Herman Tournaye, Willem Verpoest, Frederik Jan Hes, Christophe Blockeel, Claudia Spits, The interplay between mitochondrial DNA genotypes, female infertility, ovarian response, and mutagenesis in oocytes, *Human Reproduction Open*, Volume 2025, Issue 1, 2025, https://doi.org/10.1093/hropen/hoae074.

The authors would like to apologise for a minor error in the percentage of variants shown in Figure 3b. The corrected Figure  3, which includes an updated panel for Figure 3b, is shown below.

The corresponding text describing Figure 3b in the Results has also been updated. This originally erroneously stated:

*The highest load of heteroplasmic variants was observed for NonSyn variations in the protein-coding region (27%), while variants in the HV (2%), OHR (2%), and NonCoding region (2%) were observed at lower loads (Fig. 3a and b).*

It should be:

*The highest mean load of heteroplasmic variants was observed for variants in the HV and OHR region, as well as for NonSyn variations in the protein-coding region (all 1.8%). Syn variants appeared at a mean load of 1.3%, NonCod variants at 0.8% and variants in rRNA and tRNA regions at 0.6% and 0.2% respectively (Fig. 3a and b).*

The authors would like to assure readers that this error does not affect any conclusions of the article. The electronic version of this article has been updated at https://doi.org/10.1093/hropen/hoae074.

**Figure 3.**
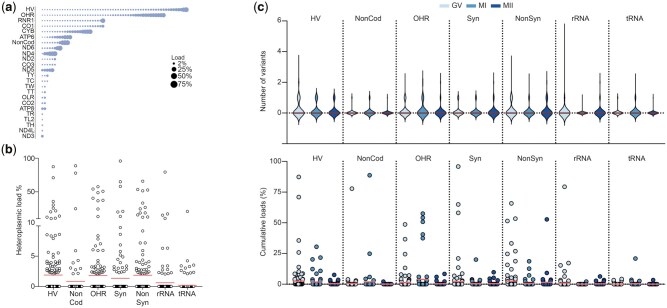
**The maturation stage of oocytes does not influence the number and load of heteroplasmic variants in the oocyte**. (a) Overview of all heteroplasmic variants identified, plotted according to their location in the mitochondrial genome. The size of the bubble is proportional to the variant’s frequency in the study population. The detection threshold was 2%. (b) Overview of the load of all heteroplasmic variants identified, categorized per region. The red line indicates the mean load. (c) Distribution of the number and load of heteroplasmic variants across the different stages of oocyte maturation. HV, hypervariable region, NonCod, non-coding region; OHR, origin of replication on the heavy strand; Syn, synonymous variant in protein-coding genes; NonSyn, non-synonymous variant in protein-coding genes; GV, germinal vesicle; MI, metaphase I; MII, metaphase II.

