# Supplementary material for: The interplay between mitochondrial DNA genotypes, female infertility, ovarian response, and mutagenesis in oocytes
Source: Hum Reprod Open. 2024 Dec 30;2025(1):hoae074. doi: 10.1093/hropen/hoae074 (PMC11739621; doi:10.1093/hropen/hoae074)
Supplement: hoae074_Supplementary_Data [file hoae074_supplementary_data.zip › Supplementary_Fig_Table-EO.docx]

Supplementary data to “**The interplay between mitochondrial DNA genotypes, female infertility, ovarian response, and mutagenesis in oocytes**”

**
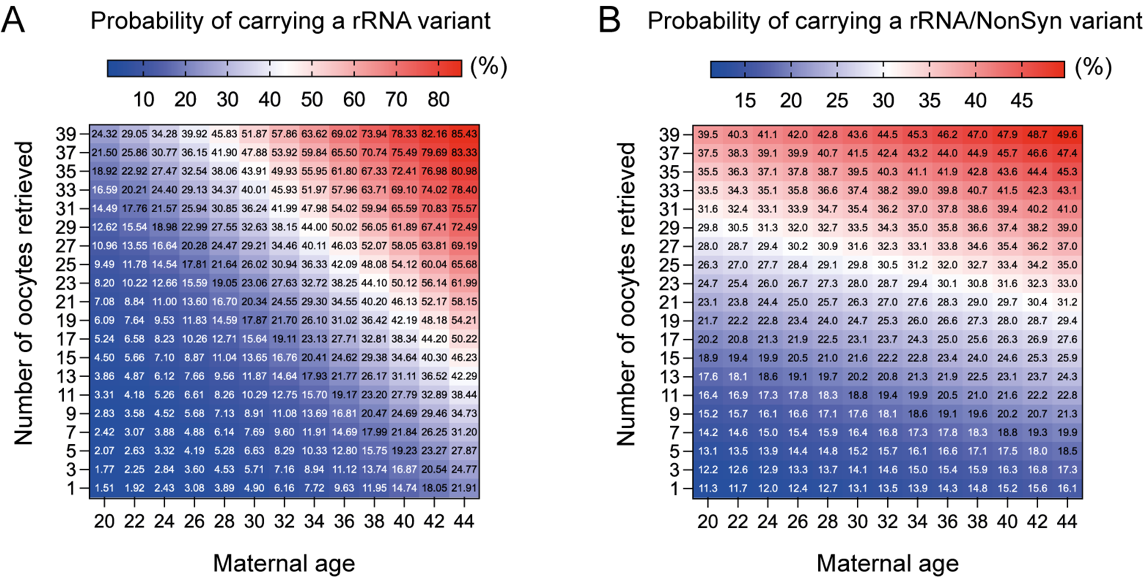
**

**Supplementary Figure S1. Probability of carrying a heteroplasmic variant in an oocyte retrieved after ovarian stimulation. A.** Heatmap depicting the probability of carrying a heteroplasmic ribosomal RNA (rRNA) variant in an oocyte retrieved after ovarian stimulation, based on maternal age and the number of oocytes retrieved after ovarian stimulation. **B.** Heatmap depicting the probability of carrying a heteroplasmic variant in either the rRNA region or a non-synonymous (NonSyn) variant in the protein coding region, in an oocyte retrieved after ovarian stimulation and based on maternal age and the number of oocytes retrieved. Color-coding ranges from blue (lower probability) to red (higher probability). Values are obtained from the regression models shown in Table 3.

**Supplementary Table S1.** Generalized Poisson loglinear regression including the female age, the total FSH stimulation units and the number of oocytes retrieved to predict the number of heteroplasmic variants in the oocytes.

|  | **B** | **95% C.I. for B** | **Significance** | **Omnibus significance** |
| --- | --- | --- | --- | --- |
| **Total number** |  | | | **0.04** |
| Female age | 0.021 | −0.011; 0.053 | 0.20 |  |
| Stim. Units | −0.00008 | 0.000; 0.000 | 0.48 |  |
| COC | 0.0081 | 0.017; 0.049 | **<0.001** |  |
| **HV** |  | | | **0.04** |
| Female age | −0.012 | −0.074; 0.049 | 0.69 |  |
| Stim. Units | 0.000 | 0.000; 0.001 | 0.33 |  |
| COC | 0.050 | 0.021; 0.080 | **<0.001** |  |
| **NonCod** |  | | | 0.14 |
| Female age | 0.024 | −0.120; 0.168 | 0.75 |  |
| Stim. Units | 0.000 | −0.001; 0.001 | 0.71 |  |
| COC | −0.025 | −0.121; 0.071 | 0.61 |  |
| **OHR** |  | | | **0.04** |
| Female age | 0.044 | −0.030; 0.118 | 0.24 |  |
| Stim. Units | 0.000 | −0.001; 0.000 | 0.09 |  |
| COC | 0.021 | −0.018; 0.060 | 0.30 |  |
| **Syn** |  | | | 0.09 |
| Female age | 0.026 | −0.068; 0.120 | 0.59 |  |
| Stim. Units | 0.000 | −0.001; 0.000 | 0.15 |  |
| COC | 0.038 | −0.008; 0.084 | 0.10 |  |
| **NonSyn** |  | | | 0.26 |
| Female age | −0.035 | −0.102; 0.033 | 0.31 |  |
| Stim. Units | 0.000 | 0.000; 0.001 | 0.32 |  |
| COC | 0.019 | −0.020; 0.057 | 0.34 |  |
| **rRNA** |  | | | **<0.001** |
| Female age | 0.121 | 0.013; 0.229 | **0.03** |  |
| Stim. Units | 0.000 | −0.001; 0.000 | 0.11 |  |
| COC | 0.080 | 0.041; 0.118 | **<0.001** |  |
| **tRNA** |  | | | 0.28 |
| Female age | 0.017 | −0.118; 0.152 | 0.80 |  |
| Stim. Units | 0.000 | −0.001; 0.001 | 0.98 |  |
| COC | −0.068 | −0.174; 0.037 | 0.20 |  |
| **NonSyn + rRNA** |  | | | **0.02** |
| Female age | 0.017 | −0.050; 0.344 | 0.55 |  |
| Stim. Units | 0.000 | 0.000; 0.000 | 0.93 |  |
| COC | 0.043 | 0.016; 0.070 | **<0.01** |  |

P-values from the generalized Poisson loglinear regression analysis < 0.05 are presented in bold. C.I., confidence interval; Stim.Units, stimulation units; HV hypervariable region; NonCod, noncoding regions; OHR, origin of heavy strand replication; Syn, synonymous variant in protein-coding genes; NonSyn, non-synonymous variant in protein-coding genes; rRNA, ribosomal RNA genes; tRNA, transfer RNA genes.

**Supplementary Table S2.** Generalized linear regression includes the female age, the total FSH stimulation units and the number of oocytes retrieved to predict the heteroplasmic load of the different variants.

|  | **B** | **95% C.I. for B** | **Significance** | **Omnibus significance** |
| --- | --- | --- | --- | --- |
| **All variants** |  | | | 0.80 |
| Female age | 0.002 | −0.004; 0.008 | 0.56 |  |
| Stim. Units | 1.609E−6 | 1.609E-6; 1.609E−6 | 0.93 |  |
| COC | 0.000 | −0.004; 0.004 | 0.78 |  |
| **HV** |  | | | 0.68 |
| Female age | 0.000 | −0.002; 0.002 | 0.84 |  |
| Stim. Units | −1.090E−6 | −1.090E−6; −1.090E−6 | 0.89 |  |
| COC | 0.001 | −0.001; 0.003 | 0.24 |  |
| **NonCod** |  | | | 0.62 |
| Female age | 0.001 | −0.001; 0.003 | 0.40 |  |
| Stim. Units | 4.392E−6 | −4.392E−6; 4.392E−6 | 0.53 |  |
| COC | −0.001 | −0.003; 0.001 | 0.28 |  |
| **OHR** |  | | | 0.38 |
| Female age | −0.001 | −0.003; 0.001 | 0.63 |  |
| Stim. Units | −3.784E−6 | −3.784E−6; 3.784E−6 | 0.62 |  |
| COC | 0.000 | −0.002; 0.002 | 0.49 |  |
| **Syn** |  | | | 0.90 |
| Female age | 0.001 | −0.001; 0.003 | 0.44 |  |
| Stim. Units | −1.057E−5 | −1.057E−5; 1.057E−5 | 0.18 |  |
| COC | 0.000 | −0.002; 0.002 | 0.82 |  |
| **NonSyn** |  | | | 0.83 |
| Female age | 0.000 | −0.002; 0.002 | 0.73 |  |
| Stim. Units | 1.540E−5 | −1.540E−5; 1.540E−5 | 0.04 |  |
| COC | 0.000 | −0.002; 0.002 | 0.48 |  |
| **rRNA** |  | | | 0.30 |
| Female age | 0.001 | −0.001; 0.003 | 0.22 |  |
| Stim. Units | −2.081E−6 | −2.081E−6; 2.081E−6 | 0.67 |  |
| COC | 0.001 | 0.001; 0.001 | 0.17 |  |
| **tRNA** |  | | | 0.87 |
| Female age | 0.000 | 0.000; 0.000 | 0.46 |  |
| Stim. Units | −2.808E−7 | −2.808E−7; 2.808E−7 | 0.83 |  |
| COC | −8.852E−5 | −8.852E−5; 8.852E−5 | 0.48 |  |

C.I., confidence interval; Stim.Units, stimulation units; HV, hypervariable region; NonCod, noncoding regions; OHR, origin of heavy strand replication; Syn, synonymous variant in protein-coding genes; NonSyn, non-synonymous variant in protein-coding genes; rRNA, ribosomal RNA genes; tRNA, transfer RNA genes.
